# Community-Engaged Research & Two-Eyed Seeing: Creating a Culturally Appropriate Dementia Screening Toolkit

**DOI:** 10.1093/geroni/igaf122.713

**Published:** 2025-12-31

**Authors:** Melissa Blind, Kandyce Garcia, January Johnson, Cassandra Thomas, Carey Gleason, Tassy Parker, Kristen Jacklin, Sheamus Cavanaugh

**Affiliations:** University of Minnesota Medical School, Duluth Campus, Duluth, Minnesota, United States; Center For Native American Health, University of New Mexico Health Sciences Center, Albuquerque, New Mexico, United States; University of Minnesota Medical School Memory Keepers Medical Discovery Team, Duluth, Minnesota, United States; WI Alzheimer’s Disease Research Center, UW Madison School of Medicine and Public Health, Madison, Wisconsin, United States; University of Wisconsin–Madison, Madison, Wisconsin, United States; University of New Mexico, Albuquerque, New Mexico, United States; University of Minnesota Medical School, Duluth Campus, Duluth, Minnesota, United States; University of Minnesota Medical School, Duluth Campus, Duluth, Minnesota, United States

## Abstract

In this paper, we present our framework for community-engaged research in our American Indigenous Cognitive Assessment (AMICA) project (R01AG074231). AMICA aims to develop and validate a culturally appropriate dementia screening toolkit, which includes a cognitive assessment, caregiver report, depression scale, and inventory of Activities of Daily Living. Our multi-site project involves community partners in Red Lake Nation, Minnesota; an urban Indigenous population in Albuquerque, New Mexico; and the Oneida Nation in Wisconsin. Each site includes local community-based researchers and Indigenous Knowledge Advisory Groups (IKAGs) that review and adapt dementia screening tools to be more culturally safe and relevant. In order to develop a single dementia evaluation toolkit for Indigenous populations throughout the United States, two IKAG members from each partner site were nominated to form a National IKAG to come to consensus on the adapted tools. Our methodology also includes the integration and use of Two-Eyed Seeing as an iterative framework, which incorporates both biomedical and Indigenous ways of knowing. In addition to the National IKAG, the AMICA project also includes an Assessment Expert Panel (AEP) consisting of experts in medical anthropology, geriatric medicine, neuropsychology, and other disciplines. We implement our community-engaged research framework to create a single intercultural dementia screening toolkit for Indigenous populations throughout the United States.

